# Comparison on self-determination, peer-relationship, and alienation in physical education of early adolescent in Korea and China

**DOI:** 10.3389/fpsyg.2024.1417914

**Published:** 2024-12-10

**Authors:** Jeong Jaeuk, Sun Yu, Suh Donghwi

**Affiliations:** ^1^Department of Physical Education, Seoul National University, Seoul, Republic of Korea; ^2^Department of Physical Education, Jeonbuk National University, Jeonju, Republic of Korea

**Keywords:** self-determination, peer-relationship, physical education alienation, early adolescent, East Asia

## Abstract

**Introduction:**

In order to identify effective strategies for enhancing a high-quality physical education system, it is imperative to undertake research that examines the nuances of educational culture, specifically focusing on the psychological experiences of early adolescents within physical education classes. This study aims to compare self-determination, peer-relationships, and feelings of alienation in physical education among adolescents from Korea and China, both situated in East Asia, with respect to their nationality and gender.

**Methods:**

One hundred and twenty two early adolescent males (*M* = 14.34, SD = 0.65) and 78 females (*M* = 14.34, SD = 0.64) from South Korea and 125 early adolescent males (*M* = 14.13, SD = 0.58) and 70 females (*M* = 14.13, SD = 0.59) from China participated in this study, with a mean age of 14.24 ± 0.62. The Academic Self-Regulatory Scale (SRQ-A), Peer-relationship Questionnaire, and a Scale for Measuring Student Alienation in Physical Education were utilized for data analysis. For statistic comparisons, a *t*-test was used.

**Results:**

Self-determination of male and female students in China was significantly higher than that of male and female students in Korea, respectively. Peer-relationship of male students in Korea was significantly higher than that of male students in China. Physical education alienation of the female students in Korea was significantly higher than that of male students in Korea.

**Discussion:**

Given that the influence of self-determination, peer relationships, and physical education alienation varies by nationality and gender, it is crucial to seek and implement strategies to develop an effective physical education system. By doing so, physical education alienation can be minimized and prevented, ultimately preserving the health and well-being of adolescents. This approach is vital for fostering a supportive and inclusive environment that promotes lifelong fitness and health.

## Introduction

World Health Organization (WHO) has emphasized that physical, social, and mental health are all essential for human health and happiness (Breslow, 1972). Hence, countries implement various systems to manage public health (Haldane et al., 2021). Adolescence marks a significant stage in human development, and governments support this development through compulsory education. Among these, physical education plays a crucial role in promoting overall physical, social, and mental well-being (Wallhead and Buckworth, 2004). Numerous studies have shown that regular physical activity improves cardiovascular health, enhances muscular strength, and promotes mental well-being by reducing symptoms of anxiety and depression. Additionally, physical activity fosters social skills and teamwork, contributing to overall social development. For instance, research has demonstrated that adolescents who engage in regular physical activity exhibit better academic performance and higher self-esteem compared to their less active peers (Calfas and Taylor, 1994). These benefits highlight the critical role of exercise in supporting the holistic health and well-being of adolescents. However, ineffective physical education classes can lead to feelings of alienation (Hascher and Hadjar, 2018). This feelings of alienation in learning discourages healthy development among students (Taylor and Adelman, 2020). In general, alienation is defined as a situation in which an individual is not integrated into their relationship with society and feels detached from the overall atmosphere of the classroom, including both pedagogical and sociological aspects (Tolbert et al., 2022). When alienation occurs within the context of physical education, it is referred to as physical education alienation. This term describes a persistent experience of negative emotions that students actively seek to avoid during physical education classes. Various factors lead to the physical education alienation, including environmental factors within the physical education setting, factors related to physical education teachers, factors related to students, and factors related to parents and the surrounding community (Lee et al., 2021). Physical education alienation, especially from the viewpoint of students, is characterized by feelings of pointlessness, helplessness, and isolation as components of the alienation experience (Spencer-Cavaliere and Rintoul, 2012). Hence, physical education alienation is directly influenced by psychological factors inherent in the learners themselves. As physical education classes primarily involve adolescents, it is important to examine various factors that are aligned with the characteristics of adolescents.

Early adolescence is marked by the development of self-identity, a transition from egocentric thinking, and an increasing awareness of others (Kroger, 2006). This age range corresponds to middle school students in Korea and China, typically aged 11–13 (Salmela-Aro, 2011). Early adolescents engage in meaningful interactions with their peers and experience social growth, often by comparing themselves to friends of similar age and developmental stage (Mitic et al., 2021). As a result, various aspects of adolescent psychology develop. Peer relationships refer to interpersonal relationships with peers and have a significant influence on the emotional and social development of adolescents (Brown and Bakken, 2011). Therefore, it is necessary to examine the psychological factor of peer-relationships in the context of the physical education alienation among early adolescents. Peer-relationships encompass sub-factors such as adaptability to peers and sustained interactions, both of which exert significant influence on the social psychological factors of adolescents (Berry et al., 1989; Mikami et al., 2010).

Adolescents, as they observe and learn from their peers, also demonstrate autonomy by applying acquired knowledge. The behavior resulting from this autonomy is referred to as self-determination. The self-determination theory (SDT) posits that comprehending human motivation necessitates acknowledging innate psychological needs for competence, autonomy, and relatedness (Ryan and Deci, 2022). Self-determination theory suggests that higher levels of self-determination are linked with better learning and achievement outcomes, as well as positive psychological effects (Gamble et al., 2023). Conversely, low levels of self-determination can result in weakened intrinsic motivation (Tang et al., 2020) and experiencing negative emotions, potentially leading to feelings of alienation in the classroom (Perlman, 2012). This implies that emotions tied to self-determination are linked with feelings of alienation within the community (Green, 2010). Self-determination is evaluated using a continuum that ranges from external motivation to internal motivation. The level of self-determination is determined using the Academic Self-Regulatory Scale (Levesque et al., 2004).

As mentioned earlier, alienation from physical education significantly impacts peer-relationships and self-determination in adolescents. Unlike other subjects, physical education classes often take the form of team sports, and even individual sports are typically conducted in the proximity of a gymnasium, which inevitably increases awareness of peers. Additionally, in physical education classes, differentiated instruction based on learners’ levels is implemented even when teaching the same sport or movement, and various roles are assigned according to individual responsiveness. Therefore, self-determination becomes a crucial factor in physical education classes (Ward et al., 2008).

However, there is limited research exploring physical education alienation, a direct and significant negative psychological factor, in conjunction with the primary characteristics of adolescents. Furthermore, adopting a cross-cultural perspective in research allows for broader insights into what is considered typical and atypical within each culture, as well as facilitates the examination of the universality of concepts (McMullen et al., 2005). Since the outbreak of the Covid-19 pandemic worldwide, research has been conducted on the psychological changes in adolescents within the educational cultures of various countries (Leon-Zarceno et al., 2021; Maugeri et al., 2020; Okuyama et al., 2021; Varea et al., 2022). China was the first country where Covid-19 originated, and Korea is its neighboring country. Analyzing the psychological changes based on the differences in educational cultures between these two nations becomes a crucial social task in preparing future generations. Korean culture places a higher value on interpersonal harmony compared to Chinese culture (Houri et al., 2012; Wadsworth et al., 2008). However, in the context of physical education, perceived peer-relationships have been found to have a greater influence in China compared to Korea (Chen and Lin, 2016). Additionally, cultural differences among members of society may lead to variations in self-determination, and in group-based physical education classes, self-determination and peer relationships can influence feelings of alienation (Kreber, 1998; Lee et al., 2000). Therefore, analyzing the differences in peer-relationships, self-determination, and feelings of alienation in physical education classes resulting from the differing cultures of Korea and China can provide valuable insights into the structure of each country’s physical education curriculum. Currently, Korea’s physical education curriculum aims to cultivate intrinsic values such as character, whereas China’s curriculum focuses on the development of athletic skills and theoretical knowledge in physical education (Xin et al., 2019). After all, Both Korea and China are striving to enhance their competitiveness in the global market, and education plays a pivotal role in achieving this goal. Excellent education is achieved when school education is developed with consideration for self-determination and cultural differences (Guay et al., 2008). Therefore, there is a need for research to analyze the differences in educational culture between the two countries in order to identify ways to further develop superior education systems. Hence, examining the cultural differences between China and its neighboring country, South Korea, particularly regarding the alienation and self-determination of adolescents in physical education classes, as well as their peer-relationships, is noteworthy. This refers to the psychological state experienced by East Asian adolescents in physical education classes. Such analysis could serve as a guide for future directions in physical education for adolescents in each country. Furthermore, in physical education, the construction an educational system that considers gender is essential due to physical differences. Therefore, there is a need to investigate the psychological states in physical education based on gender in both countries (Yamauchi et al., 2007).

This study focuses on analyzing the psychological aspects of physical education classes among East Asian adolescents, as described above. It aims to summarize and analyze previous research content to delineate future research problems, backgrounds, and research directions in the post-COVID-19 era. In particular, it will provide important data for understanding the content, issues, and students’ perceptions of physical education in Korea and China by analyzing how research directions are influenced by the characteristics of the educational environments in both countries. Therefore, the purpose of this study was to compare self-determination, peer-relationships, and alienation in physical education among middle school students from Korea and China, based on gender. This study aims to provide information and insights necessary for designing and planning physical education classes that can promote the health of East Asian adolescents in the post-COVID-19 era. It will achieve this by comparing the self-determination, peer-relationships, and alienation experienced by adolescents in physical education classes, taking into account the cultural context of East Asia.

## Research method

### Research subjects

In this study, the participants consisted of 200 middle school students (122 males: 14.34 ± 0.65; 78 females: 14.34 ± 0.64) from three schools in South Korea and 195 middle school students (125 males: 14.13 ± 0.58; 70 females: 14.13 ± 0.59) from three schools in China (see Table 1). The required number of subjects was calculated using the G*Power 3.1 program (Effect size: 0.5; α: 0.05; power: 0.80; Total sample size:128). The effect size of 0.5 indicates a medium effect according to Cohen’s conventions, which suggests a meaningful difference between the groups being compared. The adopted cut-off values for interpreting the effect sizes were set at 0.2 for small, 0.5 for medium, and 0.8 for large effects, allowing for a clear understanding of the practical significance of the findings. The students attend different middle schools in each country, and three urban schools in both Korea and China were sampled using cluster sampling with stratification. All participants and their parents were asked to read and sign an written informed consent form. This study was approved by local ethical committee (IRB No. 2024-07-040-001). The study was conducted in accordance with the Declaration of Helsinki.

**Table 1:** Participants of middle school students from Korea and China.

National	Gender		Grade			Total
	Male	Female	1	2	3	
Korea (age)	N:122 (14.34 ± 0.65)	N:78 (14.34 ± 0.64)	N:19 (13)	N:94 (14)	N:87 (15)	N:200 (14.34 ± 0.64)
China (age)	N:125 (14.13 ± 0.58)	N:70 (14.13 ± 0.59)	N:22 (13)	N:125 (14)	N:48 (15)	N:195 (14.13 ± 0.58)
Total (age)	N:247 (14.24 ± 0.63)	N:148 (14.24 ± 0.62)	N:41 (13)	N:219 (14)	N:135 (15)	N:395 (14.24 ± 0.62)

### Measurement tool

#### Self-determination

The questionnaire used to measure self-determination type is derived from the Academic Self-Regulatory Scale (SRQ-A) developed by Levesque et al. (2004). This questionnaire was designed to categorize participants’ motivational types based on their level of self-determination. A total of 29 sub-factors, consisting of 29 questions, were partially modified and supplemented (see Supplementary Table S1). In adapting and enhancing the Academic Self-Regulatory Scale, initial modifications involved tailoring item phrasing to better fit the context of this study’s target population. Specific adjustments were made to clarify terminology and improve question relevance based on pilot testing feedback. Additionally, supplementary questions were incorporated to expand upon the original sub-factors, allowing for a more nuanced assessment of self-determination types. Following these modifications, reliability analysis was conducted to ensure the validity of the scale. This study utilized a questionnaire grounded in the conceptualization of self-determination theory to identify learners’ self-determination types (Deci and Ryan, 2004). The questionnaire employs a six-point Likert scale, ranging from “not at all” (1 point), “disagree” (2 points), “neutral” (3 points), “somewhat agree” (4 points), “agree” (5 points), to “strongly agree” (6 points), assessing levels of asynchrony, extrinsic motivation, imposition, and identification. The five subtypes of regulation and internal regulation are encompassed. The reliability analysis yielded a Cronbach’s α value of 0.893. Furthermore, the exploratory factor analysis revealed satisfactory results (KMO = 0.871; Bartlett’s test of sphericity, $X^{2}$ = 8353.082, *p* < 0.001), indicating duplicated items. Ultimately, 20 items representing 3 factors were extracted. Detailed results are presented in Supplementary Table S2. The development of the experimental tools was overseen by two Ph.D. holders in sports psychology.

#### Peer relationship

To assess the relationship between students, the peer relationship questionnaire was adapted and enhanced (Berry et al., 1989). This scale comprises 20 questions across 4 sub-factors, including friendship, adaptation, trust with friends, and communal life with friends. The questionnaire employs a six-point Likert scale, ranging from “not at all” (1 point), “disagree” (2 points), “neutral” (3 points), “somewhat agree” (4 points), “agree” (5 points), to “strongly agree” (6 points). In adapting and enhancing the peer relationship questionnaire, an initial review of the original items was conducted to ensure cultural relevance and appropriateness for the target population. Modifications were made to align the questions with the specific characteristics of the student demographic. This involved rephrasing certain items for clarity and incorporating feedback from pilot testing to refine question wording and structure. Furthermore, reliability analysis was conducted to identify redundant or low-performing items. Based on these findings, exploratory factor analysis was used to confirm the structure of the questionnaire and to retain only items that demonstrated strong factor loadings, thus ensuring the scale’s validity and reliability for the final assessment. Following exploratory factor analysis (KMO = 0.753; Bartlett’s test of sphericity, $X^{2}$ = 1596.583, *p* < 0.001), duplicated items were identified. Ultimately, 10 items representing 3 factors were extracted (Supplementary Table S3).

#### Physical education alienation

To assess physical education alienation, the questionnaire titled ‘Development of Scale for Measuring Student Alienation in Physical Education’ was utilized, with consultation from two Ph.D. holders in sports psychology (Kim, 2005). The questionnaire was adapted and augmented to be appropriate for middle school students in both China and Korea. To adapt and enhance the original scale for middle school students in China and Korea, initial adjustments were made to ensure cultural relevance and age-appropriateness. This process involved rephrasing certain items to improve clarity and readability for early adolescents. Additionally, supplementary items were incorporated to capture a broader range of alienation experiences specific to the physical education context. Feedback from a pilot study informed further refinements, allowing for the removal of ambiguous or redundant questions. Following these modifications, exploratory factor analysis was conducted to confirm the structure and eliminate duplicated or low-performing items, resulting in a refined scale that accurately represents the key dimensions of physical education alienation. The scale comprises seven questions each on physical education alienation and extracurricular activities, along with six questions on athletic ability alienation, friend alienation, and sports alienation, respectively. The questionnaire employs a six-point Likert scale, ranging from “not at all” (1 point), “disagree” (2 points), “neutral” (3 points), “somewhat agree” (4 points), “agree” (5 points), to “strongly agree” (6 points). Following exploratory factor analysis (KMO = 0.736; Bartlett’s test of sphericity, $X^{2}$ = 3571.202, *p* < 0.001), duplicated questions were identified. Ultimately, 18 questions representing 5 factors were extracted. Specific results are presented in Supplementary Table S4.

### Research procedure

The questionnaire for this study was distributed directly to the research participants by their physical education teacher. The questionnaires were administered during March 2024. The data were collected through a questionnaire comprising questions about three variables, which were presented sequentially and collected all at once. Students were allotted approximately 15 min to complete the questionnaire, which contained 53 questions.

### Data processing

After the questionnaires were collected, they were sorted and assigned numbers. The data were coded and analyzed using SPSS 23.0. The validity and reliability of the questionnaires were assessed through exploratory factor analysis and Cronbach’s alpha test. The main analytical methods utilized in this study comprised descriptive statistics, independent samples *t*-tests, and stepwise multiple regression analysis. Differences were analyzed using *t*-tests based on the country variable, with a significance level set at 0.05.

## Results

To investigate the differences in self-determination, peer-relationships, and physical education alienation among middle school students in Korea and China based on gender and country, this study employed independent sample *t*-tests. Significant differences were detected in self-determination, peer-relationships, and physical education alienation between genders and countries. As depicted in Table 2, the level of self-determination among Korean students was significantly lower than that among Chinese students (*p* < 0.001). Additionally, both Korean male and female students exhibited significantly lower levels of self-determination compared to their Chinese counterparts (*p* < 0.001 for both male and female students). The gender differences were as follows: self-determination among male Korean students was significantly higher than among female Korean students (*p* < 0.05). However, among Chinese students, there was no significant difference in self-determination based on gender.

**Table 2:** Self-determination according to country and gender.

Country	Korean male (*n* = 122) Korean female (*n* = 78) Korea total (*n* = 200)		Chinese male (*n* = 125) Chinese female (*n* = 70) Chinese total (*n* = 195)			
	*M*	SD	*M*	SD	*t*	Cohen’s d
Male	65.369	15.943	74.504	16.182	−4.450^***^	−0.566
Female	60.231	14.908	71.200	15.529	−4.352^***^	−0.717
Total	63.365	15.748	73.318	16.029	−6.209^***^	−0.625
Gender	Korean male (*n* = 122)Chinese male (*n* = 125)		Korean female (*n* = 78)Korean female (*n* = 78)			
	*M*	SD	*M*	SD	*t*	Cohen’s d
Korean	65.369	15.943	60.231	14.908	2.268^*^	0.329
Chinese	74.504	16.182	71.200	15.529	1.380	0.209

***p < 0.001, *p < 0.05.

As depicted in Table 3, the peer-relationship among total Korean students was significantly higher than that among Chinese students (*p* < 0.001). According to gender, only male Korean students exhibited significantly higher peer-relationships than male Chinese students (*p* < 0.001). The gender differences were as follows: There were no differences in peer-relationships among Korean students based on gender. However, among Chinese students, male students exhibited significantly lower peer-relationships compared to female Chinese students (*p* < 0.001).

**Table 3:** Peer-relationship according to country and gender.

Country	Korean male (*n* = 122) Korean female (*n* = 78) Korea total (*n* = 200)		Chinese male (*n* = 125) Chinese female (*n* = 70) Chinese total (*n* = 195)			
	*M*	SD	*M*	SD	*t*	Cohen’s d
Male	24.951	5.629	22.176	5.607	3.881^***^	0.388
Female	26.487	5.167	25.271	6.474	1.260	0.207
Total	25.550	5.505	23.287	6.116	3.858^***^	0.388
Gender	Korean male (*n* = 122)Chinese male (*n* = 125)		Korean female (*n* = 78)Korean female (*n* = 78)			
	*M*	SD	*M*	SD	*t*	Cohen’s d
Korean	24.951	5.629	26.487	5.167	−1.933	−0.280
Chinese	22.176	5.607	25.271	6.474	−5.463^***^	−0.565

***p < 0.001, *p < 0.05.

As depicted in Table 4, there were no significant differences in physical education alienation between the two countries. The gender differences were as follows: Physical education alienation among male Korean students was significantly lower than among female Korean students (*p* < 0.05). However, among Chinese students, there was no significant difference in physical education alienation based on gender.

**Table 4:** P.E. Alienation according to country and gender.

Country	Korean male (*n* = 122) Korean female (*n* = 78) Korea total (*n* = 200)		Chinese male (*n* = 125) Chinese female (*n* = 70) Chinese total (*n* = 195)			
	*M*	SD	*M*	SD	*t*	Cohen’s d
Male	43.844	10.689	43.512	8.097	0.275	0.035
Female	47.167	7.565	45.100	9.075	1.500	0.247
Total	45.140	9.753	44.082	8.517	1.147	0.115
Gender	Korean male (*n* = 122)Chinese male (*n* = 125)		Korean female (*n* = 78)Korean female (*n* = 78)			
	*M*	SD	*M*	SD	*t*	Cohen’s d
Korean	43.844	10.689	47.167	7.565	−2.378^*^	−0.345
Chinese	43.512	8.097	45.100	9.075	−1.251	−0.187

*p < 0.05.

## Discussion

In summary, Chinese students scored higher in self-determination than Korean students for both male and female groups. For peer-relationships, Korean male students scored higher than Chinese male students, while in terms of P.E. alienation, Korean female students scored higher than Korean male students. Effect sizes were calculated for all comparisons to provide a better understanding of the practical significance of the results. Cohen’s d was used to assess the magnitude of differences, with cut-off values following Cohen’s guidelines: small effects (*d* = 0.2), medium effects (*d* = 0.5), and large effects (*d* = 0.8). For each significant finding, Cohen’s d value was calculated and presented in the tables (Tables 2–4). In this study, effect sizes ranged from −0.717 to 0.388. The negative effect size values indicate that, for certain comparisons, the mean of one group was lower than the other, while positive values indicate the opposite. These results suggest mostly small to medium effects. This indicates that while statistical significance was achieved, the practical significance varies depending on the strength and direction of the observed effect sizes (Schäfer and Schwarz, 2019).

When comparing between the two countries, the self-determination of Chinese students was higher than that of Korean students, while the peer-relationship of Korean students was higher than that of Chinese students. This is likely attributed to previous research findings indicating differences in the types of sports experienced by Korean and Chinese students, as well as lower levels of interpersonal relationships among Chinese students compared to Korean students (Houri et al., 2012; Wadsworth et al., 2008). In other words, Korean adolescents who prioritize interpersonal harmony tend to have higher levels of peer-relationships, whereas Chinese adolescents who prioritize individual values tend to have higher levels of self-determination. This could be interpreted as a reflection of national cultural differences concerning interpersonal relationships (Blanchard et al., 2009; Gay, 2013).

Additionally, Korea has a curriculum designed to foster core competencies such as character through physical activity (Xin et al., 2019). Therefore, it is possible that peer-relationships, which are considered one of the core competencies, appear higher among Korean students compared to Chinese students. In China, a curriculum is implemented to cultivate core competencies through athletic skills and health knowledge. Therefore, it can be interpreted that there is a high level of self-determination among Chinese students as they strive to improve their athletic skills and health knowledge. From this perspective, it could be inferred that implementing physical education classes emphasizing self-determination may be effective for students in Chinese culture, while promoting peer-relationships may be beneficial for students in Korean culture, which emphasizes mutual learning styles (Morgan, 2017).

About gender differences in Korea, male students exhibit higher levels of self-determination compared to female students, while female students in Korea experience higher levels of physical education alienation than their male counterparts, consistent with findings from several previous studies. This is attributed to the fact that gender stereotypes of masculinity and femininity in Korea are reflected in young adolescents’ participation in sports (You et al., 2021). This implies that physical activity and sports participation are perceived as more suitable for boys. This leads male students to develop relatively strong athletic abilities due to their physical characteristics and the social atmosphere, which fosters a sense of competence (Joong-gil, 2012). Male students in small clubs engage in active communication both within club sports and online gaming communities. Club activities foster intricate and enduring relationships not only within the campus but also beyond the school grounds (Cho and Choi, 2015). In contrast, in China, female students exhibited higher levels of peer relationships compared to male students, consistent with previous research findings. According to previous research, the low peer relationships among Chinese male students are associated with high levels of aggression (Chen et al., 2012). Therefore, given that the peer relationships of Chinese male students were relatively lower than those of Chinese female students, it is important to consider implementing sports programs in the form of team sports, which can enhance peer relationships (Danioni and Barni, 2019), and addressing issues of aggression through physical activity (Koc, 2017). As schools resume face-to-face classes following the coronavirus pandemic, it is crucial to monitor mental health through peer relationships (Zhu et al., 2022). In particular, it has been reported that peer relationships resulting from physical activity have a greater impact on academic achievement for Chinese male students compared to Chinese female students (Bi et al., 2022; Li et al., 2020). Therefore, designing physical education programs that take these variables into consideration will be effective in promoting the value of human co-prosperity through education. Thus, it is important to consider these factors when organizing physical education classes based on gender and culture. Hence, future research should investigate whether designing classes that consider various psychological factors of Korean and Chinese students, such as self-check or reciprocal styles, promote positive experiences in physical education settings (Chatoupis, 2018; Hein et al., 2012).

Given that psychological well-being and optimal functioning are associated with internal motivation, it is important to plan physical education classes with consideration of cultural characteristics such as self-determination and peer relationships, which significantly impact early adolescence (White et al., 2021). Hence, to enhance self-determination and peer relationships, it would be beneficial to actively incorporate new sports, diversify physical education programs, adjust the level of difficulty, and utilize modified games (Wang et al., 2020). It is believed that the development and supplementation of various physical education programs are necessary to alter the perception of physical education, which has traditionally been focused mainly on male students (Kwon and Block, 2017; Corr et al., 2019). Meanwhile, Jeong et al. (2014) analyzed the phenomenon of students’ alienation in physical education to explore strategies for addressing it. The causes of physical education alienation included individual physical ability, negative experiences in physical education, interpersonal factors, class content, and classroom environment. As physical education alienation impacts self-determination, it is important to ensure that physical education classes are well-structured (Kreber, 1998; Lee et al., 2000). Since higher self-determination is associated with higher social problem-solving ability (Lee et al., 2000), well-structured physical education can contribute to fostering a positive social culture.

Additionally during the COVID-19 pandemic, Korea and China implemented different policies and social distancing measures, which may have affected the experiences of adolescents in unique ways. In Korea, strict social distancing and periodic school closures might have influenced peer relationships as students had less face-to-face interaction. This could result in heightened peer-relationship scores once in-person classes resumed, as students were more eager to rebuild connections. In contrast, China’s emphasis on rapid containment measures and a focus on online learning might have encouraged students to become more self-reliant, potentially contributing to higher levels of self-determination. The varied COVID-19 measures might also have influenced how students perceive P.E. classes. Korean students, having experienced significant social limitations, may have found P.E. classes a valuable outlet for socialization, whereas Chinese students may have engaged more in individual sports during the pandemic, fostering a sense of personal achievement and autonomy. These contextual factors highlight the importance of considering how differing national responses to COVID-19 may indirectly shape adolescents’ educational and social experiences. This allowed us to comprehend the experiences of adolescents in physical education and their psychological states, influenced by the diverse cultures and societal perceptions of gender in Korea and China. This represents a novel approach to adolescent health education within the education system, considering the cultural and social contexts of East Asia in the post-COVID-19 era.

While demographic information, including grade level, preference for physical education, and weekly exercise time, was collected, analyses of these factors were not conducted in this study. The primary focus was on examining self-determination, peer relationships, and P.E. alienation among Chinese and Korean adolescents. There is also a limitation in that the sample size is too small to interpret differences in grade, preference, and exercise time as cultural differences between countries, as such demographic information may vary depending on the city within each country. Future research may consider exploring these additional demographic variables to gain deeper insights into their potential impacts on the key areas of interest.

## Conclusion

The findings of this study reveal significant differences in self-determination, peer-relationships, and feelings of alienation among adolescents in South Korea and China. First, Chinese adolescents demonstrated higher levels of self-determination, suggesting that China’s physical education programs, which emphasize personal value and growth, should continue to promote autonomy and self-direction among students. Second, South Korean adolescents exhibited stronger peer-relationships, reflecting a cultural emphasis on socialization and interpersonal harmony in their physical education. South Korea should leverage this strength to foster positive peer interactions further. Lastly, feelings of alienation were lower among South Korean male adolescents compared to their female counterparts, highlighting the need for gender-specific approaches in educational programs.

Overall, this research underscores the importance of adapting physical education programs in both countries to reflect their cultural contexts and meet the needs of adolescents. Educators should recognize the strengths and weaknesses of their respective programs to provide a healthy and inclusive physical education experience for all students. This will serve as foundational data for establishing an educational environment that fosters the physical, mental, and social well-being of youth, as defined by the World Health Organization, who will be the leaders of the future.

## Data Availability

The original contributions presented in the study are included in the article/Supplementary material, further inquiries can be directed to the corresponding author.
