# Supplementary material for: Comparison on self-determination, peer-relationship, and alienation in physical education of early adolescent in Korea and China
Source: Front Psychol. 2024 Dec 10;15:1417914. doi: 10.3389/fpsyg.2024.1417914 (PMC11668143; doi:10.3389/fpsyg.2024.1417914)
Supplement: Supplementary file 1 [file Table_1.docx]

**Supplementary tables**

**Table S1. Configuration of Questionnaire**

| Contents | Dimensions | Question | n |
| --- | --- | --- | --- |
| Demographics | Country, Gender, Grade, Preference, Weekly exercise time | 1,2,3,4,5 | 5 |
| Self-determination | Intrinsic-Identified,  Interjected,  External | 1,2,3,4,5,6,7,8,9,10  11,12,13,14,15,16  17,18,19,20 | 10  6  4 |
| Peer-relationship | Adaptability-reliability,  Life with-adaptability,  Companion Continuity | 1,2,3,4  5,6,7  8,9,10 | 4  3  3 |
| Physical education alienation | Friend  Facilities  P.E. teacher-energy  Curriculum  Sportswear | 1,2,3,4  5,6,7  8,9,10,11,12,13  14,15,16  17,18 | 4  3  6  3  2 |
| Total |  |  | 53 |
