# Supplementary material for: Comparison on self-determination, peer-relationship, and alienation in physical education of early adolescent in Korea and China
Source: Front Psychol. 2024 Dec 10;15:1417914. doi: 10.3389/fpsyg.2024.1417914 (PMC11668143; doi:10.3389/fpsyg.2024.1417914)
Supplement: Supplementary file 2 [file Table_2.docx]

**Supplementary tables**

**Table S2. Self-determination of exploratory factor analysis results (Korea-China)**

| Subfactor | Ingredient | | |
| --- | --- | --- | --- |
|  | Intrinsic-Identified | Interjected | External |
| Intrinsic motivation2  Intrinsic motivation3  Intrinsic motivation5  Intrinsic motivation4  Intrinsic regulation3  Intrinsic regulation5  Intrinsic motivation1  Intrinsic regulation2  Intrinsic regulation4  Intrinsic regulation1 | .931  .905  .905  .899  .860  .858  .847  .824  .807  .734 | .006  -.074  -.041  .022  .053  .100  .108  .102  .150  .353 | -.066  -.187  -.196  -.106  -.034  -.089  -.023  -.012  .045  .022 |
| Interjected regulation5  Interjected regulation2  Interjected regulation3  Interjected regulation1  Interjected regulation6  Interjected regulation4 | .121  -.079  .096  -.030  .204  .150 | .916  .882  .868  .858  .831  .773 | .079  .102  .233  .182  -.028  .093 |
| External regulation3  External regulation2  External regulation1  External regulation6 | -.103  .114  -.207  -.109 | .108  .186  .020  .164 | .828  .820  .759  .667 |
| Eigenvalue  Variance (%)  Cumulative variance (%)  Reliability (loyal .893) | 7.550  37.749  37.749  .962 | 4.657  23.286  61.036  .932 | 2.595  12.977  74.013  .774 |
