# Supplementary material for: Comparison on self-determination, peer-relationship, and alienation in physical education of early adolescent in Korea and China
Source: Front Psychol. 2024 Dec 10;15:1417914. doi: 10.3389/fpsyg.2024.1417914 (PMC11668143; doi:10.3389/fpsyg.2024.1417914)
Supplement: Supplementary file 3 [file Table_3.docx]

**Supplementary tables**

**Table S3. Exploratory factor analysis results of Peer-relationship (Korea-China)**

| Subfactor | Ingredient | | |
| --- | --- | --- | --- |
|  | Adaptability-reliability | Life with-adaptability | Companion Continuity |
| Friend adaptability2  Friend adaptability1  Friendliness reliability4  Friendliness reliability2 | .859  .819  .701  .597 | .029  .086  -.208  -.071 | .008  .213  .311  .318 |
| Life with friends3  Life with friends2  Friend adaptability5 | .050  .020  -.156 | .910  .855  .766 | -.088  -.216  .161 |
| Companion continuity4  Companion continuity5  Companion continuity3 | .271  .234  .135 | -.130  -.251  .190 | .827  .803  .698 |
| Eigenvalue  Variance (%)  Cumulative variance (%)  Reliability (loyal .753) | 2.431  24.314  24.314  .757 | 2.318  23.185  47.499  .730 | 2.139  21.393  78.892  .809 |
