# Supplementary material for: Comparison on self-determination, peer-relationship, and alienation in physical education of early adolescent in Korea and China
Source: Front Psychol. 2024 Dec 10;15:1417914. doi: 10.3389/fpsyg.2024.1417914 (PMC11668143; doi:10.3389/fpsyg.2024.1417914)
Supplement: Supplementary file 4 [file Table_4.docx]

**Supplementary tables**

**Table S4. Exploratory factor analysis results of physical education alienation (Korea-China)**

| Subfactor | Ingredient | | | | |
| --- | --- | --- | --- | --- | --- |
|  | Friend | Facilities | P.E. teacher-energy | Curriculum | Sportswear |
| Friend relationship2  Friend relationship3  Friend relationship4  Friend relationship5 | .837  .776  .760  .704 | .136  -.104  .210  .207 | .080  .307  .123  .272 | .104  -.153  -.108  -.159 | .004  .109  .164  .243 |
| Sports facilities4  Sports facilities2  Sports facilities1 | .209  .047  .077 | .886  .886  .838 | -.016  .131  -.007 | -.113  .017  -.030 | -.032  .087  -.058 |
| P.E. teacher7  P.E. teacher2  P.E. teacher3  P.E. teacher1  Sports energy5 | -.033  .154  .206  .366  .265 | .217  .075  -.100  -.139  .121 | .763  .744  .739  .586  .575 | -.159  -.208  .130  .005  -.372 | -.016  .105  -.035  .041  .130 |
| Curriculum7  Curriculum1  Curriculum5 | -.119  -.027  -.030 | .049  .049  -.362 | -.184  -.068  -.045 | .879  .878  .792 | .046  -.103  .266 |
| Sportswear2  Sportswear4 | .171  .151 | .059  -.073 | .088  .015 | .023  .061 | .916  .912 |
| Eigenvalue  Variance (%)  Cumulative variance (%)  Reliability (loyal .753) | 2.767  16.275  16.275  .840 | 2.629  15.465  31.741  .867 | 2.611  15.361  47.102  .774 | 2.483  14.606  61.708  .844 | 1.896  11.150  72.858  .854 |
